# A Novel Anti-Inflammatory Role for Ginkgolide B in Asthma via Inhibition of the ERK/MAPK Signaling Pathway

**DOI:** 10.3390/molecules16097634

**Published:** 2011-09-06

**Authors:** Xiao Chu, Xinxin Ci, Jiakang He, Miaomiao Wei, Xiaofeng Yang, Qingjun Cao, Hongyu Li, Shuang Guan, Yanhong Deng, Daxin Pang, Xuming Deng

**Affiliations:** 1 Key Laboratory of Zoonosis Ministry of Education, Institute of Zoonosis, College of Animal Science and Veterinary Medicine, Jilin University, Changchun 130062, China; 2 College of Animal Science and Technology, Guangxi University, 100 Daxue Road, Nanning 530005, Guangxi, China; 3 College of Plant Science, Jilin University, Changchun 130062, China; 4 Key Laboratory of Animal Embryo Engineering, Department of Animal Biotechnology, College of Animal Science and Veterinary Medicine, Jilin University, Changchun 130062, China

**Keywords:** ginkgolide B, MAPK, asthma, airway inflammation, airway hyper-responsiveness

## Abstract

Ginkgolide B is an anti-inflammatory extract of *Ginkgo biloba* and has been used therapeutically. It is a known inhibitor of platelet activating factor (PAF), which is important in the pathogenesis of asthma. Here, a non-infectious mouse model of asthma is used to evaluate the anti-inflammatory capacity of ginkgolide B (GKB) and characterize the interaction of GKB with the mitogen activated protein kinase (MAPK) pathway. BALB/c mice that were sensitized and challenged to ovalbumin (OVA) were treated with GKB (40 mg/kg) one hour before they were challenged with OVA. Our study demonstrated that GKB may effectively inhibit the increase of T-helper 2 cytokines, such as interleukin (IL)-5 and IL-13 in bronchoalveolar lavage fluid (BALF). Furthermore, the eosinophil count in BALF significantly decreased after treatment of GKB when compared with the OVA-challenged group. Histological studies demonstrated that GKB substantially inhibited OVA-induced eosinophilia in lung tissue and mucus hyper-secretion by goblet cells in the airway. These results suggest that ginkgolide B may be useful for the treatment of asthma and its efficacy is related to suppression of extracellular regulating kinase/MAPK pathway.

## 1. Introduction

Asthma is a complex disease characterized by acute and chronic airway inflammation, airway hyper-responsiveness (AHR), eosinophilia and mucus hypersecretion by goblet cells. Many cytokines contribute to this inflammation mediated by T-helper 2 (Th2) cells, which play central roles in the pathogenesis of allergic asthma [1,2]. Interleukin (IL)-5 expressed by Th2 cells, is responsible for eosinophil growth, differentiation, mobilization, recruitment, activation, and survival [3,4,5]. Interleukin (IL)-13 play an important role in T-cell differentiation toward a Th2 phenotype and isotype switching of B cells to immunoglobulin IgE production [6,7]. Interleukin (IL)-13 promotes acute inflammatory processes and underlying structural changes to the airways [8]. Thus, antagonizing the action of Th2-type cytokines represents one of the major new therapeutic strategies in the treatment of bronchial asthma. 

The morbidity and mortality of asthma appear to be increasing, and it has been suggested that medications used to treat asthma, that is Chinese Materia Medica, are contributing to this trend. *Ginkgo biloba* has been used as an herb in traditional Chinese medicine for thousands of years. Ginkgolide B (GKB), the major active component of *G. biloba* extracts, is a known inhibitor of platelet activating factor (PAF), which is important in the pathogenesis of asthma [9]. GKB primarily induces activation of intracellular signaling events and has the potential to prime cellular functions such as PMN defense activities [10], and induces apoptosis via activation of c-Jun N-terminal kinase (JNK) and p21-activated protein kinase 2 in mouse embryonic stem cells [11]. Ginkgolides offer a desirable approach for this due to their low toxicity [11]. Moreover, Tosaki A *et al.* showed that *G. biloba* extract can improve contractile function after global ischemia in the isolated working rat heart by reducing the formation of oxygen free radicals [12].

The mitogen activated protein kinases (MAPKs) are evolutionary conserved enzymes which play a key role in signal transduction mediated by cytokines, growth factors, neurotransmitters and various types of environmental stresses. The MAPK family includes three distinct stress-activated protein kinase pathways: p38, JNK, and extracellular regulating kinase (ERK) [13]. It has been reported that inhibition of the MAPK signalling pathway in lung inflammatory cells (e.g., mast cells) may have therapeutic potential in the treatment of allergic diseases such as asthma [14]. Based on studies investigating the effect of GKB, however, no available study has been done in a mouse model of allergic airway inflammation, so we focused on investigating whether GKB possesses a distinct anti-inflammatory activity on a non-infectious mouse model of asthma, and elucidated the involvement with MAPK pathway for the first time.

## 2. Results and Discussion

### 2.1. GKB Reduces Ovalbumin-induced Bronchoalveolar Lavage Fluid T Helper Type 2 Cytokine Levels

**Figure 1:**
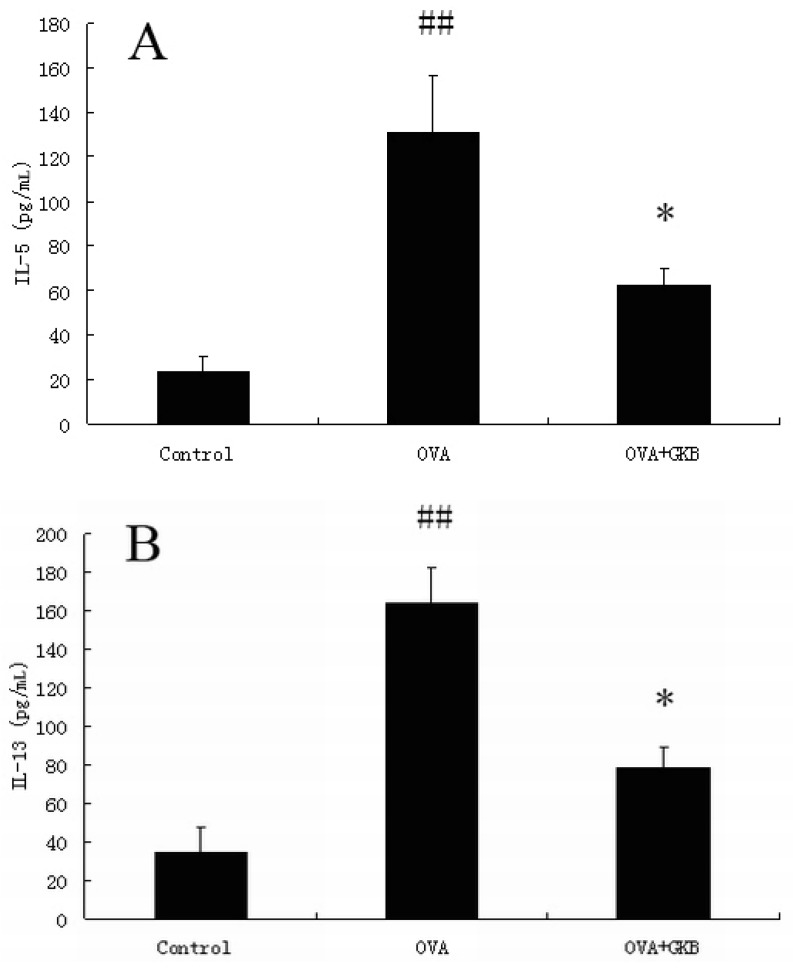
Effects of ginkgolide B on the secretion of IL-5 and IL-13. The lavage fluid was centrifuged, and the supernatants were measured by ELISA. The values represent the means ± SEM of three independent experiments. GKB = ginkgolide B. (^##^ p < 0.01 *vs.* control group mice, * p < 0.05 *vs.* OVA-challenged mice).

Th2 cytokines levels in the bronchoalveolar lavage were measured by a sandwich ELISA. The concentrations of IL-5 and IL-13 were increased in OVA-immunized samples compared to control mice (Figure 1). Treatment with GKB caused a reduction in the levels of IL-5 and IL-13 compared to ovalbumin-immunized mice (Figure 1).

### 2.2. GKB Reduces OVA-Induced Serum Levels of OVA-specific IgE

**Figure 2:**
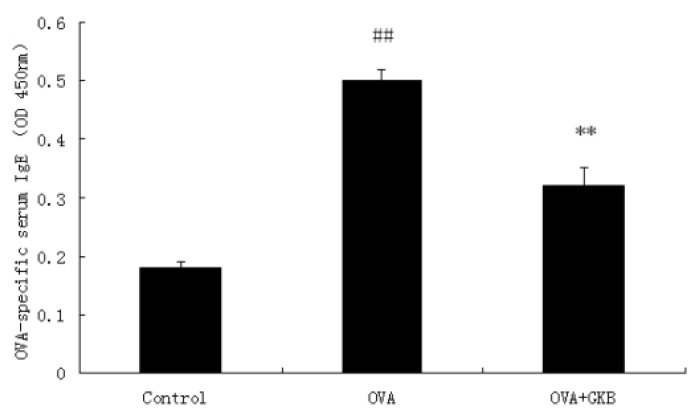
Effects of ginkgolide B on OVA-specific IgE in serum. OVA-specific IgE levels in the serum were measured by ELISA. Results (means ± SEM) are expressed as Optical Density values and are representative of at least three independent experiments, GKB = ginkgolide-B ^(##^ p < 0.01 *vs.* control group mice, ** p < 0.01 *vs.* OVA-challenged mice).

OVA-induced serum levels of OVA-specific IgE were analyzed by a sandwich enzyme-linked immunosorbent assay. OVA-immunized mice treated with a vehicle had high levels of serum anti- OVA IgE antibodies compared to control mice (Figure 2). A significant reduction in OVA-specific IgE antibodies was observed in mice treated with GKB (Figure 2).

### 2.3. GKB Reduces OVA-Induced Bronchoalveolar Lavage Fluid (BALF) Inflammatory Cell Recruitment

**Figure 3:**
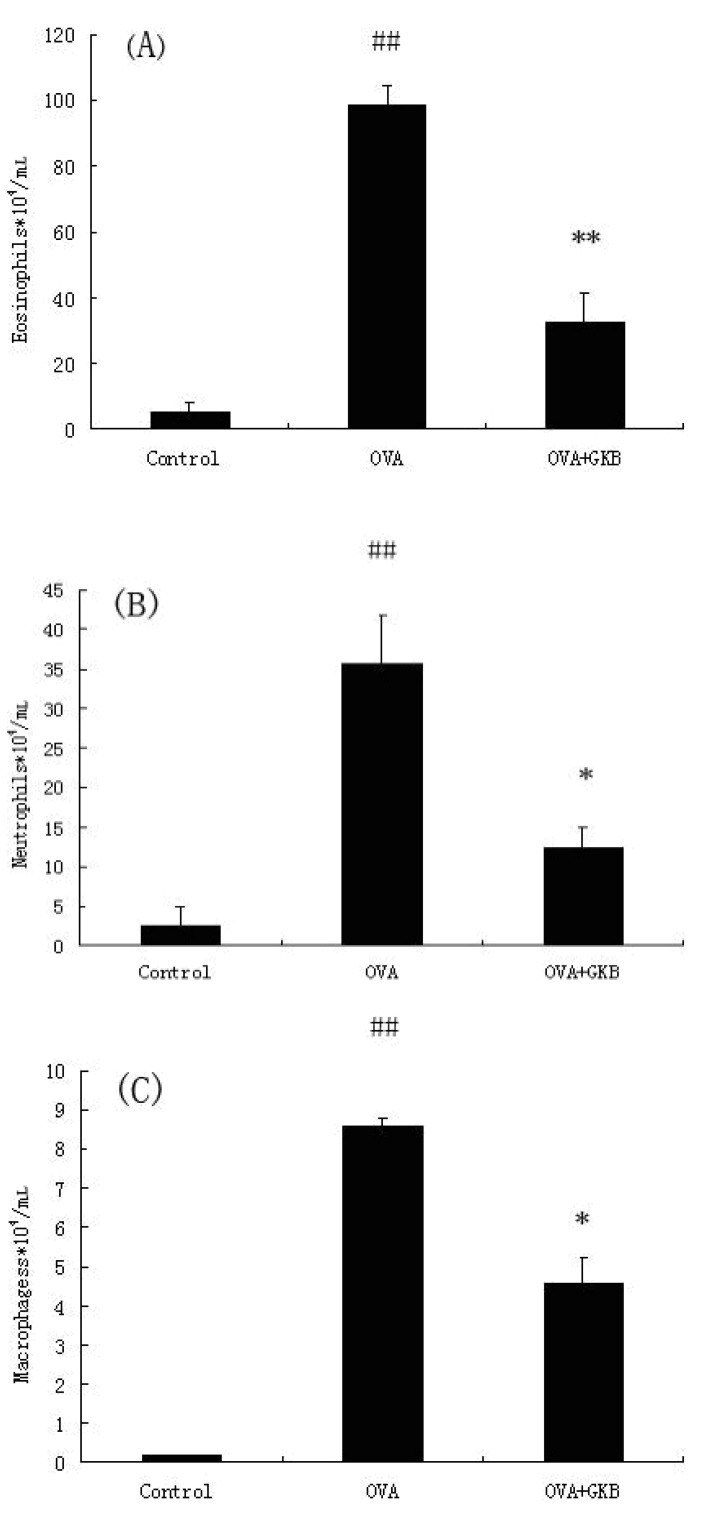
Effects of ginkgolide-B on the recruitment of inflammatory cell in BALF. The lavage fluid was centrifuged, and the cell pellets were resuspended and applied to a slide by cytospinning to obtain differential cell counts by staining with a modified Giemsa method. The values represent the means ± SEM of three independent experiments. GKB = ginkgolide-B (^##^ p < 0.01 *vs.* control group mice, * p < 0.05, ** p < 0.01 *vs.* OVA-challenged mice).

The total cell counts and differential cell counts in the BALF were evaluated 24 h after the last OVA challenge. As shown in Figure 3, OVA-immunized mice treated with a vehicle had higher levels of eosinophils, neutrophils, and macrophages compared to the control group. However, GKB significantly decreased the number of eosinophils, neutrophils, and macrophages (Figure 3).

### 2.4. Effects of GKB on OVA-Induced Airway Hyper-Responsiveness

**Figure 4:**
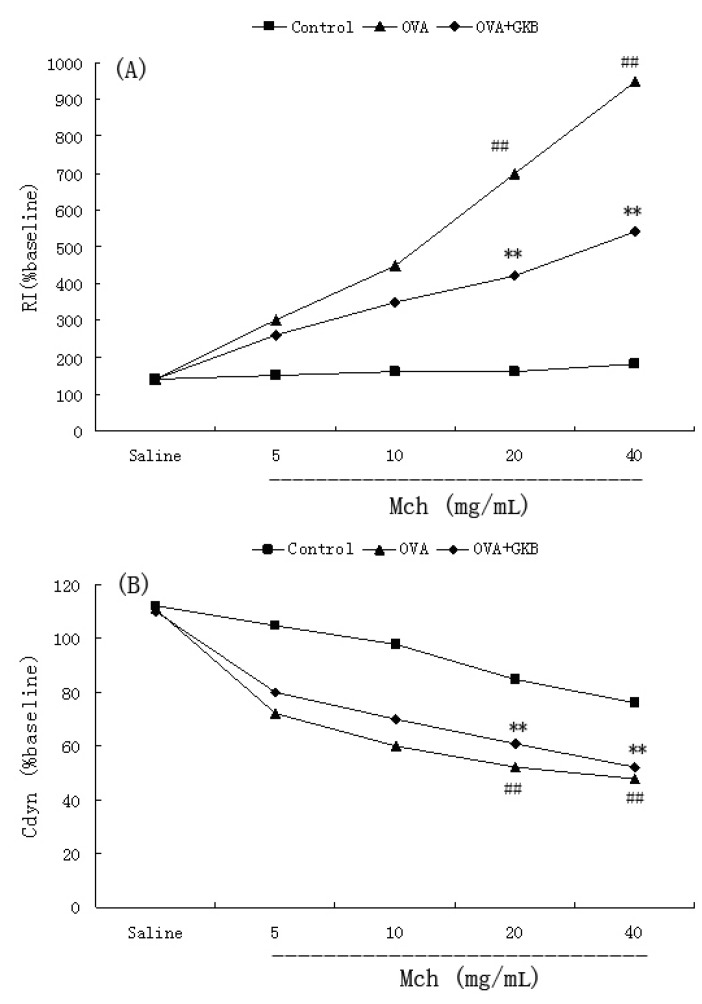
Effects of ginkgolide-B on OVA-induced airway hyper-responsiveness in mice. Airway hyper-responsiveness was assessed by percentage change from the baseline level of (A) lung resistance (RI, n = 10 mice per treatment group) and (B) dynamic compliance (Cdyn, n =10 mice per treatment group). GKB = ginkgolide-B, Mch = Methacholine (^##^ p < 0.01 *vs.* control group mice, ** p < 0.01 *vs.* OVA-challenged mice).

To investigate the effect of GKB on AHR in response to increasing concentrations of methacholine, we measured both RI and Cdyn in mechanically ventilated mice. OVA-challenged mice developed AHR, as was typically reflected by a high RI and low Cdyn (Figure 4). GKB treatment significantly reduced RI and restored Cdyn in OVA-challenged mice in response to methacholine (Figure 4).

### 2.5. Effects of GKB on OVA-Induced Airway Goblet Cell Hyperplasia and Mucus Production

**Figure 5:**
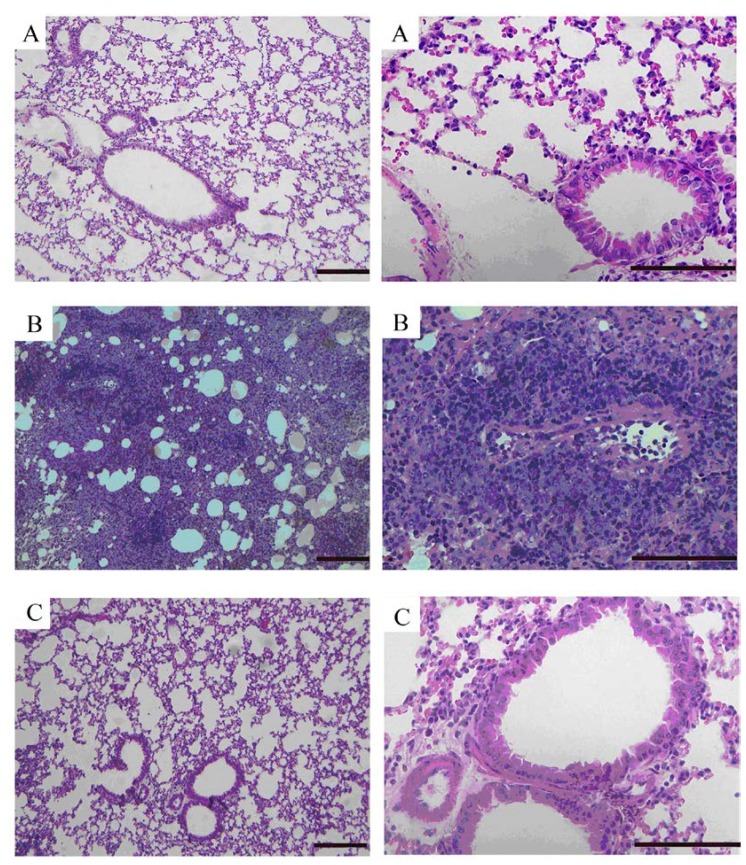
Effects of ginkgolide B on airway inflammation. Representative haematoxylin-eosin stained sections of lung from: (**A**) PBS-challenged mice; (**B**) OVA-challenged mice; (**C**) OVA-challenged mice treated with ginkgolide B. The left panel is magnified × 100, scale bars = 100 μm. The right panel is magnified × 400, scale bars = 50 μm.

**Figure 6:**
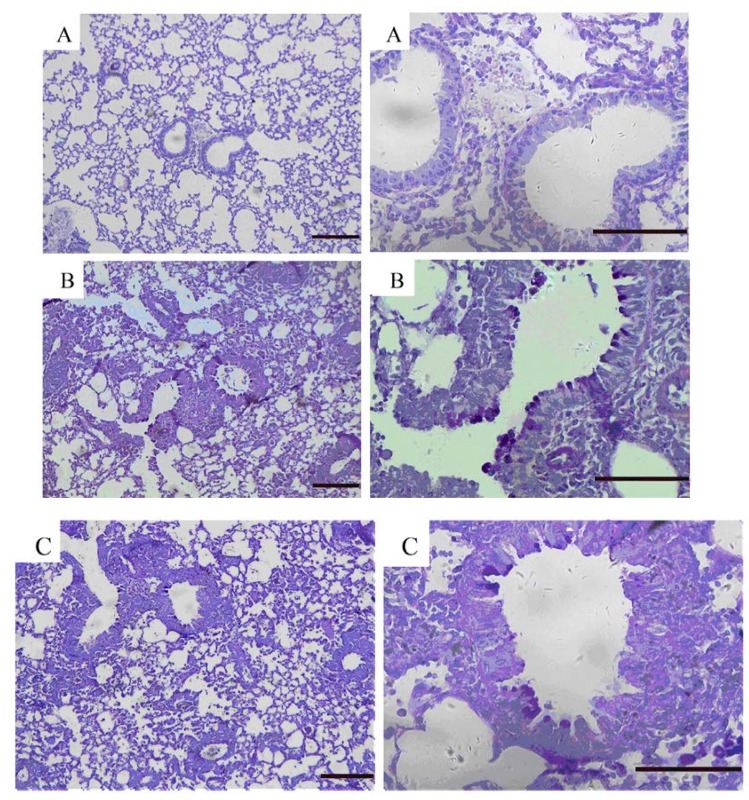
Effects of ginkgolide B on airway goblet cell hyperplasia and mucus production. Representative alcian blue-periodic acid-Schiff stained sections of lung from: (**A**) PBS-challenged mice; (**B**) OVA-challenged mice; (**C**) OVA-challenged mice treated with ginkgolide B. The left panel is magnified × 100, scale bars = 100 μm. The right panel is magnified × 400, scale bars = 50 μm.

To evaluate the effect of GKB on airway inflammation, airway goblet cell hyperplasia and mucus production. We stained lung tissues with haematoxylin-eosin (Figure 5) and alcian blue-periodic acid-Schiff (Figure 6) staining solutions to examine the inhibitory effect of GKB on the histological change in the OVA-induced asthma model.

The lungs of the OVA-induced mice showed dense perivascular and peribronchial infiltration of leukocytes and mucus secretion within the bronchi when compared to the control tissue. GKB treatment significantly attenuated airway inflammation (Figure 5), mucus secretion and goblet cell hyperplasia (Figure 6).

**Figure 7:**
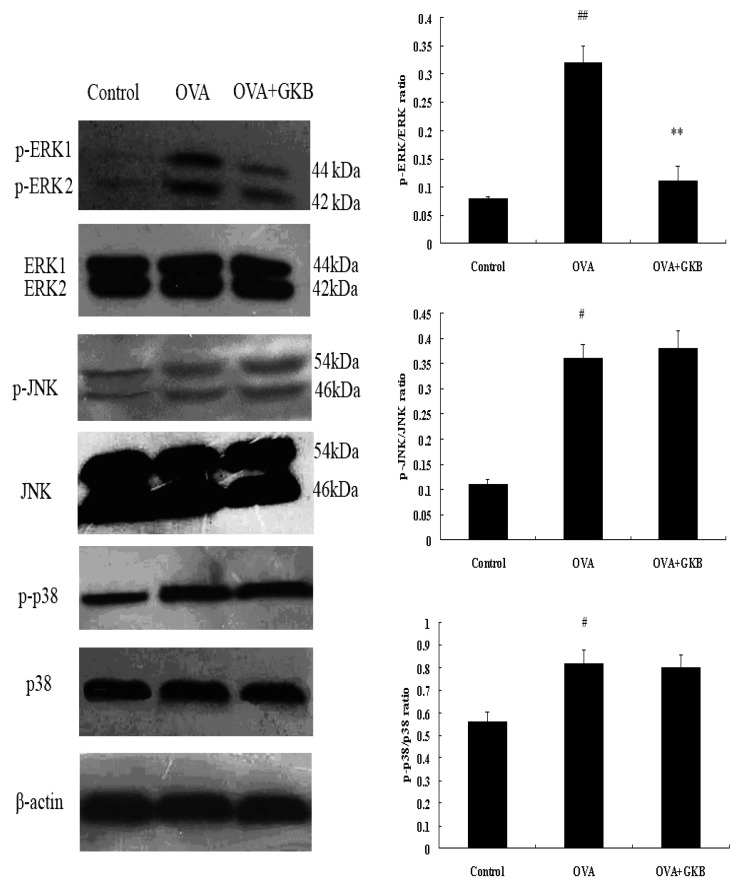
Effects of ginkgolide B on MAPK activation *in vivo*. Total cellular proteins from lung were analyzed by Western blot with specific antibodies. Experiments were repeated three times and similar results were obtained, n = 10 mice per treatment group (^#^ p < 0.05, ^##^ p < 0.01 *vs.* control group mice, ** p < 0.01 *vs.* OVA-challenged mice).

### 2.6. Effects of GKB on Activation of p38, ERK and JNK

Our data showed that p38, ERK, and JNK were activated after the last OVA challenge. Treatment with GKB significantly inhibited the activation of ERK/MAPK compared to OVA-challenged mice. However, there was no significant change in p-JNK and p-p38 between OVA-challenged mice and the group treated with GKB (Figure 7). These results showed that GKB exert its anti-inflammatory actions via inhibition of ERK/MAPK signaling pathway.

### 2.7. Discussion

Allergic asthma is a chronic airway inflammation disease. In most asthma phenotypes, increases in eosinophil levels are observed in the tissues, blood, and bronchoalveolar lavage ﬂuid. Furthermore, Th2 cells and their secreted products aggregate into airway and lung tissues. In addition, high serum levels of immunoglobulin E (IgE), persistent airway AHR, and goblet cell hyperplasia are observed. Ginkgolide B is a component of traditional Chinese herbal medicines. It improves cardiac function after ischaemia in both non-preconditioned and preconditioned non-diabetic and diabetic rats [15]. The combinations of *Ginkgo biloba* leaf extract (EGb761) plus the carotenoid antioxidant astaxanthin (ASX) and vitamin C are evaluated for summative dose effect in inhibition of asthma associated inflammation in asthmatic guinea pigs [16]. Tosaki *et al.* demonstrated that the combination of superoxide dismutase (SOD), catalase and EGB 761 may synergistically reduce the formation of free radicals and the incidence of reperfusion-induced VF and VT [17]. However, this is the first time the anti-inflammation and AHR-inhibiting effect of GKB is demonstrated in a mouse model of bronchial asthma. We also investigated the association with the MAPK pathway. Our results suggest that GKB may be used as a therapeutic reagent for patients with allergic airway inflammation.

Th2 cells are essential for the pathogenesis of asthma. Numerous studies have established a critical function for the Th2 cytokines IL-5 and IL-13 in the asthmatic response. The growth, activation, and survival of eosinophils are associated with IL-5 [18,19], and with the help of IL-13, it regulates eosinophil trafficking into sites of inflammation [20,21]. IL-13 control eosinophil trafficking directly by up-regulating adhesion molecules on endothelial cells [22] or by inducing chemokine expression in the airway. In the present study, the expression of IL-5 and IL-13 in lung, which was measured by a sandwich ELISA in the OVA group, was increased compared to the control group. In contrast, pretreatment with GKB resulted in a significant reduction of IL-5 and IL-13 in lung tissues. 

Recent studies have demonstrated that airway inflammation is a major contributing factor to the pathogenesis and pathobiology of allergic asthma. The levels of airway inflammation often correlate with the severity of clinical symptoms, the degree of airway obstruction, and AHR. Anti-inflammatory therapies are central to long-term asthma management. Treatment strategies aimed at normalizing surrogates of airway inflammation (e.g., sputum eosinophils and AHR) have better outcomes than solely treating the symptoms or improving lung function [23,24]. Our data demonstrated that GKB inhibited OVA-induced AHR resulting in inhaled methacholine. Meanwhile, eosinophilia aggregation into tissue was also inhibited by GKB. IL-13 has been shown to induce AHR in mouse models of asthma [25]. IL-5-mediated eosinophilia contributes to AHR by generating cytotoxic products [26]. Therefore, the inhibition of AHR by GKB may be associated with the reduction of Interleukin IL-5 and IL-13 production and the eosinophilia aggregation into the lungs.

In animal models, OVA challenges induced a significant increase in the total serum IgE and BALF IgE [27,28]. Our data showed that the serum concentration of IgE was significantly reduced in allergic mice after GKB administration. This result suggests that GKB has an effect on the allergic asthma that developed in an IgE-dependent manner. 

MAPKs are highly conserved, eukaryotic signal transducing enzymes that respond to environmental stresses, as well as to plasma membrane receptor stimulation, by regulating key molecular targets, up to the transcriptional machinery in the nucleus. This enzyme family includes several subgroups such as JNK, ERK and p38. ERK signaling pathway is activated upon ligation of T cell receptor in T cells, B cell receptor in B cells, and FcεRI in mast cells, leading to proliferation, differentiation, cytokine production, and degranulation [29,30,31,32]. ERK activity in the lungs of asthmatic mice was significantly higher as compared with normal mice [33]. Duan *et al.* reports that regulation of ERK signaling pathway could modulate allergic airway inflammation [34]. In trying to understand the mechanisms by which GKB elicits its salutary effects, we investigated the effect of GKB on the MAPK. Western blot analysis showed that GKB markedly attenuated OVA-induced tyrosine phosphorylation of ERK1/2. However, there was no significant change in phosphorylation of JNK and p38 between OVA-challenged mice and the group treated with GKB. Our results showed that ERK signaling pathway plays an important role in the anti-inflammatory property of GKB in asthma model.

## 3. Experimental

### 3.1. Animals

Female BALB/c mice, weighing approximately 16 to 18 g, were purchased from the Center of Experimental Animals of Baiqiuen Medical College of Jilin University (Jilin, China). Mice were housed for 2-3 days to adapt them to the environment before experimentation. The mice were housed in micro-isolator cages and received food and water *ad libitum*. The laboratory temperature was 24 ± 1 °C, and relative humidity was 40–80%. All animal experiments were performed in accordance with the guide for the Care and Use of Laboratory Animals published by the US National Institutes of Health. 

### 3.2. Reagent

**Figure 8:**
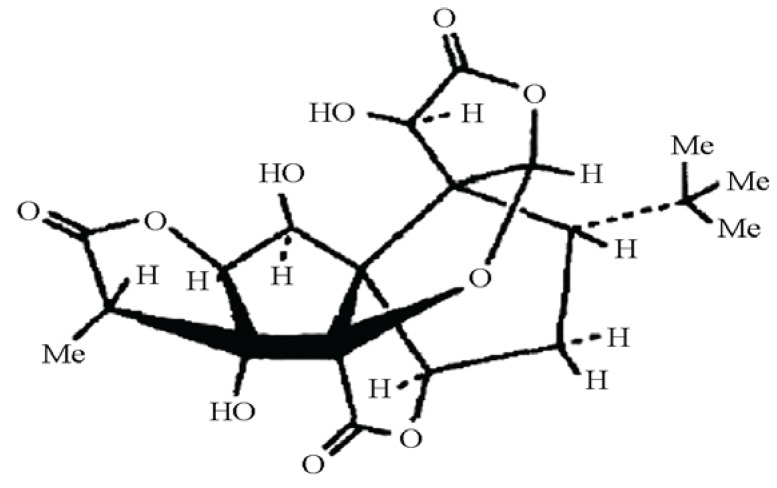
Structure of Ginkolide B.

The IL-5 and IL-13 ELISA kits were purchased from Biolegend (California, USA). Ovalbumins (Grade δ) were purchased from Sigma-Aldrich (St. Louis, MO, USA). GKB (purity >98%, Figure 8) was purchased from National Institute for the Control of Pharmaceutical and Biological Products (Beijing, China). Phospho-specific antibodies for ERK1/2, p38 and JNK as well as antibodies against ERK1/2, p38, JNK and β-actin proteins were obtained from Cell Signaling Technologies (Beverly, MA, USA). Peroxidase-conjugated Affinipure Goat Anti-Mouse IgG (H+L) and Peroxidase-conjugated Affinipure Goat Anti-Rabbit IgG (H+L) were purchased from PTG (Chicago, IL, USA). The ELISA kits for IgE were purchased from R&D (Anniston, AL, USA). The purity of all chemical reagents was at least analytical grade.

### 3.3. Antigen Sensitization, Challenge and GKB Treatment

Groups of mice (n = 10), receiving the following treatments were studied: (1) sham-sensitization plus challenge with PBS; (2) sensitization plus challenge with OVA; and (3) sensitization plus challenge with OVA and treated with GKB. Groups of 10 animals were used for each experimental condition. Mice were sensitized with OVA (20 μg) adsorbed in Imject Alum (100 μg/mL, Pierce, Rockford, IL, USA) by intraperitoneal application on days 0 and 14. On days 25-27, mice were again anesthetized, intranasally challenged with OVA (100 μg) in PBS (50 μL). The negative controls were sham-sensitized and challenged with PBS following the same protocol. GKB (40 mg/kg dissolved in PBS) was administered by intraperitoneal application at 1 h before the OVA challenge on days 25-27.

### 3.4. BALF and Serum Collection

Mice were anesthetized 24 h after the last OVA challenge and were bled via the brachial plexus to collect the blood samples that were used to estimate IgE production. Ice-cold PBS (0.5 mL) was instilled twice into the lungs, and BAL fluid was collected. Total cell counts were performed using a hemocytometer. The fluid recovered from each sample was centrifuged (4 °C, 3,000 rpm, 10 min) to pellet the cells, and the supernatant was kept at −70 °C until it was used for cytokine measurements. The cell pellets were resuspended in PBS to stain and count the total number of cells using the Wright-Giemsa staining method. At least 200 cells were counted per slide.

### 3.5. Cytokine Levels in Lung Tissues

The concentrations of cytokine IL-5 and IL-13 in the supernatants of the BALF were measured by sandwich enzyme-linked immunosorbent assay using commercially available reagents according to the manufacturer’s instructions. 

### 3.6. Mouse Anti-OVA IgE ELISA

To define serum levels of OVA-specific IgE, an ELISA analysis was carried out using a mouse-specific anti-IgE-antibody. Briefly, microplate wells were coated with 1% OVA in coating buffer (0.05 M sodium carbonate–bicarbonate, pH 9.6) overnight at 4 °C. The wells were then incubated with blocking buffer (1% BSA in PBS, pH 7.2) at room temperature for 1 h and washed. Then, the diluted (1/10) serum samples were introduced to the microplate, which was then incubated at room temperature for 2 h, washed, and incubated with Biotin anti-mouse IgE. The samples were followed by the addition of extravidin-peroxidase at room temperature for 30 min and TMB substrate for 15 min. The enzymatic reaction was stopped with 2 M H_2_SO_4_, and the absorbance was read at 450 nm. Units are reported as the optical density (OD) at 450 nm.

### 3.7. Determination of Airway Hyper-Responsiveness

Mice were anesthetized, and tracheotomy was performed as described [35]. The internal jugular vein was cannulated and connected to a microsyringe for intravenous methacholine administration. Airway resistance (RI) and lung compliance (Cdyn) in response to increasing concentrations of methacholine were recorded using a whole-body plethysmograph chamber (Buxco, Sharon, CT, USA) as described [23]. RI is defined as the pressure driving respiration divided by flow. Cdyn refers to the distensibility of the lung and is defined as the change in volume of the lung produced by a change in pressure across the lung. Results are expressed as the percentage of the respective basal values.

### 3.8. Histological Examination

Histopathologic evaluation was performed on mice that were not subjected to BALF. Left lungs were removed by dissection and fixed in 4% paraformaldehyde. Lung tissues were sectioned, embedded in paraffin, and cut at 3 μm. Tissue sections were then stained with hematoxylin and eosin (H&E) for general morphology and AB-PAS (alcian blue-periodic acid-Schiff) for the identification of goblet cells in the epithelium.

### 3.9. Western Blot Analysis

Tissues were harvested and frozen in liquid nitrogen immediately until homogenization. Samples were homogenized in RIPA buffer and lysed for 30 min on ice. Total protein fractionation was performed using a cell lysis buffer for western blot and IP (Beyotime Institute of Biotechnology, China) according to the manufacturer’s protocol. Protein concentration was assayed using the Bio-Rad protein kit, and equal amounts of protein were loaded into wells on a 10% sodium dodecyl sulphate (SDS)-polyacrylamide gel. Subsequently, proteins were transferred onto polyvinylidene difluoride (PVDF) membranes, blocked overnight with 5% (wt/vol) nonfat dry milk, and probed according to the method described by Towbin *et al*. [36]. with specific antibodies against JNK, ERK1/2, p38, β-actin antibodies, phospho-specific antibodies to JNK, ERK1/2, p38 in 5% (wt/vol) BSA dissolved in TTBS. With the use of a peroxidase-conjugated secondary anti-mouse or anti-rabbit antibody, bound antibodies were detected by ECL plus (GE Healthcare Buckinghamshire, UK).

### 3.10. Statistical Analysis

Data are presented as means ± SEM. One-way ANOVA followed by Dennett test was used to determine significant differences between treatment groups. The critical level for significance was set at p < 0.05.

## 4. Conclusions

Our study demonstrated that GKB may effectively inhibit the increase of Th2 cytokines, such as IL-5 and IL-13 in BALF. In addition, the eosinophil count in BALF was significantly decreased after treatment of GKB compared to the OVA-challenged group. Histological studies demonstrated that GKB substantially inhibited OVA-induced eosinophilia in lung tissue and mucus hyper-secretion by goblet cells in the airway. Taken together, our findings suggest that GKB may effectively inhibit the ERK signaling pathway and may serve as a therapeutic reagent for patients with allergic airway inflammation.
